# Fundamentals of Thermal Expansion and Thermal Contraction

**DOI:** 10.3390/ma10040410

**Published:** 2017-04-14

**Authors:** Zi-Kui Liu, Shun-Li Shang, Yi Wang

**Affiliations:** Department of Materials Science and Engineering, The Pennsylvania State University, University Park, PA 16802, USA; sus26@psu.edu (S.-L.S.); yuw3@psu.edu (Y.W.)

**Keywords:** negative thermal expansion (NTE), thermal contraction, theory, thermodynamics, statistical mechanics

## Abstract

Thermal expansion is an important property of substances. Its theoretical prediction has been challenging, particularly in cases the volume decreases with temperature, i.e., thermal contraction or negative thermal expansion at high temperatures. In this paper, a new theory recently developed by the authors has been reviewed and further examined in the framework of fundamental thermodynamics and statistical mechanics. Its applications to cerium with colossal thermal expansion and Fe_3_Pt with thermal contraction in certain temperature ranges are discussed. It is anticipated that this theory is not limited to volume only and can be used to predict a wide range of properties at finite temperatures.

## 1. Introduction

**Figure 1:**
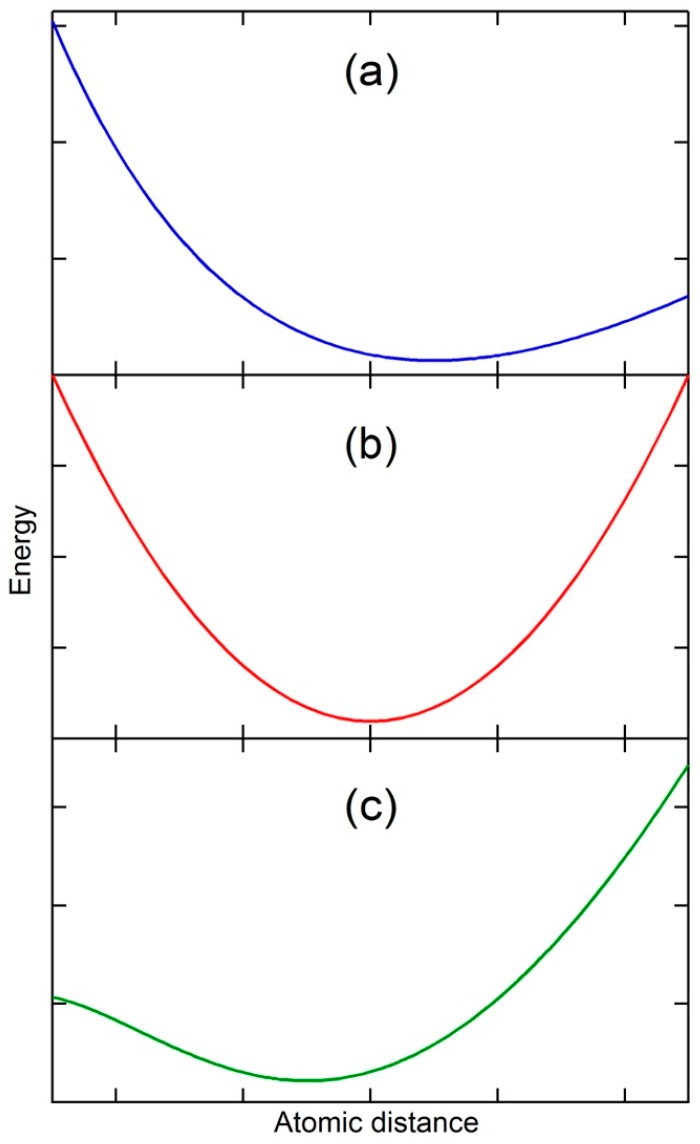
Schematic potential energy of a substance with (**a**) thermal expansion; (**b**) zero thermal expansion; (**c**) thermal contraction or negative thermal expansion.

It is well known that thermal expansion originates from the effect of anharmonic terms in the potential energy on the mean separation of atoms at a temperature [1], as schematically shown in Figure 1a in the vicinity of equilibrium separation distance. When the temperature is increased, the kinetic energy of atoms increases, and the atoms vibrate and move, resulting in a greater average separation of atoms and thus thermal expansion, i.e., the vibrational origin of thermal expansion. It is therefore self-evident that for a substance with a constant separation of atoms at a temperature, i.e., zero thermal expansion, its potential energy must be symmetric with respect to the separation (see Figure 1b). Furthermore, if the separation of the substance at a temperature decreases with the increase in temperature, i.e., thermal contraction or negative thermal expansion, its potential energy must be again asymmetric as shown in Figure 1c, but in the opposite direction of thermal expansion. The state with zero thermal expansion is thus the boundary between the thermal expansion and thermal contraction. Thermal contraction is counterintuitive as the increase in kinetic energy with temperature should push atoms apart rather then pull them together, but in reality many substances experience thermal contraction in certain temperature and pressure ranges [2].

There are a number of excellent reviews on this topic in the literature [3,4,5,6,7]. Many theoretical interpretations for thermal contraction have been developed for individual or groups of substances, but there is a lack of fundamental understanding how the potential energy of a substance can change its asymmetry into the opposite direction with increasing temperature from Figure 1a, to Figure 1b, to Figure 1c, and then back to Figure 1a,b. Consequently, thermal contraction could not be predicted with the existing theories and models, particularly at high temperatures. In our previous works [2,8,9,10], we proposed that a single-phase substance at high temperatures may consist of many states, each with its own potential energy similar to Figure 1a. All these states, except one and its degeneracies, are metastable in terms of total energy. When the statistical probabilities of the metastable states increase with temperature, and their physical properties, such as volume, may have values larger or smaller than those of the stable state, the behaviors of the substance can be significantly altered, resulting in anomalies such as thermal contraction. Furthermore, our computational approach based on first-principles calculations and statistical mechanics was able to predict the critical points of cerium and Fe_3_Pt and the associated maximum anomalies of thermal expansion in cerium and thermal contraction in Fe_3_Pt, i.e., positive and negative infinite at their critical points, respectively, in addition to thermal expansion and contraction temperature ranges in their single-phase regions under various pressures [10].

In the present paper, the fundamentals of thermal expansion and thermal contraction are discussed in terms of the asymmetry of potential energy. Our previously proposed theory is reviewed and further analyzed based on these fundamentals, and thus becomes more general and comprehensive.

## 2. Thermodynamics of a Substance

For the sake of simplicity, let us consider a closed system at equilibrium with its combined law of thermodynamics written as

(1) dG=−SdT−Vd(−P)

where *G*, *S*, and *V* are the Gibbs energy, entropy, and volume of the system, and *T* and *P* the temperature and pressure, respectively. It is noted that *S* and *T* are conjugate variables, so are *−P* and *V*. The stability of the system requires that the conjugate variables change in the same direction as follows [11,12]:

(2) $${\left(\frac{\partial T}{\partial S}\right)}_{V\;or\;P}>0$$

(3) $${\left(\frac{\partial \left(-P\right)}{\partial V}\right)}_{S\;or\;T}>0$$

When both derivatives become zero, the system reaches the critical point and becomes unstable, resulting in the maximum anomalies of entropy and volume changes as shown below:

(4) $${\left(\frac{\partial S}{\partial T}\right)}_{V\;or\;P}={\left(\frac{\partial V}{\partial \left(-P\right)}\right)}_{S\;or\;T}=+\infty$$

A positive sign is put in front of infinity because the derivatives are positive before they approach infinity as shown by Equations (2) and (3). For an unstable system, one or more of its vibrational frequencies become imaginary, commonly referred as soft vibrational modes [13].

It is evident that the derivative of volume to temperature under constant pressure, i.e., isobaric thermal expansion, at the critical point is also infinite, but there are no fundamental relations indicating whether the derivative is positive or negative because volume and temperature are not conjugate variables. What is known is the Maxwell relation derived from the second derivatives of Gibbs energy from Equation (1) as follows [11,12]:

(5) $${\left(\frac{\partial V}{\partial T}\right)}_{P}=\frac{\partial^{2}G}{\partial T\partial \left(-P\right)}=\frac{\partial^{2}G}{\partial \left(-P\right)\partial T}={\left(\frac{\partial S}{\partial \left(-P\right)}\right)}_{T}$$

It can be seen from Equation (5) that the thermal expansion is related to entropy change with respect to pressure. Even though entropy in a stable system increases with its conjugate variable, temperature, as shown by Equation (2), its relation with its non-conjugate variable, pressure, is not defined by any fundamental laws either, the same as the thermal expansion.

In Figure 1, the negative pressure, *−P*, is represented by the slope of the potential energy curve. In Figure 1a, the higher entropy at higher temperature corresponds to positive values of (*−P*) with larger separations of atoms, and *S* and (*−P*) thus vary in the same direction, resulting in positive values for Equation (5), i.e., thermal expansion. While in Figure 1c, the higher entropy at higher temperature corresponds to negative (*−P*) with smaller separations of atoms, and *S* and (*−P*) thus vary in the opposite directions, resulting in negative values for Equation (5), i.e., thermal contraction. In the next section, the transitions between potential energies shown in Figure 1 are discussed.

## 3. Potential Energy on Mean Separation of Atoms/Molecules

**Figure 2:**
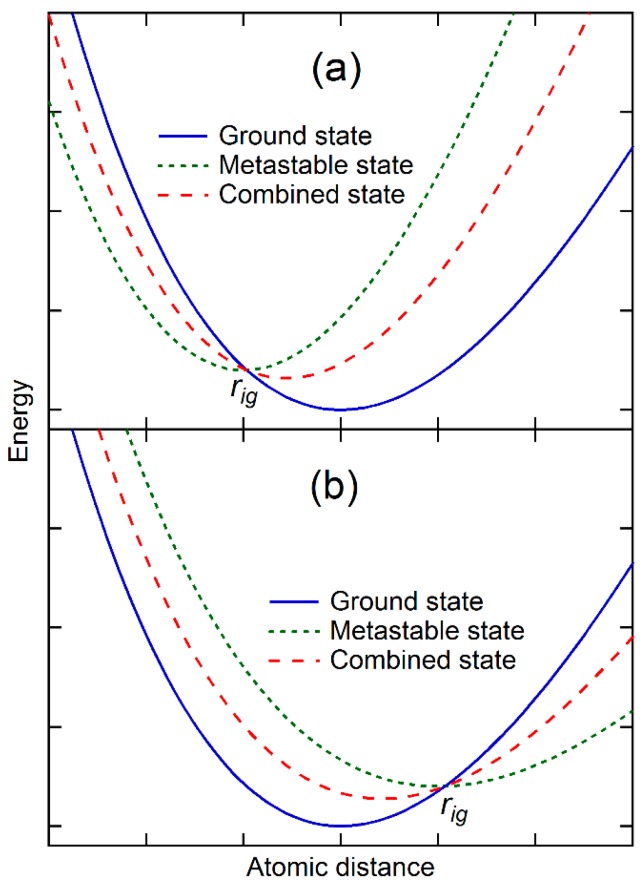
Schematic diagrams depicting the combination of potential energies of various configurations/states of a substance to show (**a**) thermal contraction or negative thermal expansion; and (**b**) thermal expansion.

For any given states of a substance, their potential energies at 0 K can be calculated from first-principles calculations based on the density functional theory (DFT) [14,15]. All exhibit shapes similar to the one shown in Figure 1a with the ground state having the lowest minimum potential energy for a classical oscillator [1], and other metastable states having higher minimum potential energies and larger or smaller equilibrium atomic/molecular distances and volumes. It is evident that the potential energy curve at the vicinities of the minimum potential energy in Figure 1b,c may be mathematically represented by the weighted average of potential energies of the ground state and those metastable states as schematically shown in Figure 2. The combined potential energy, $E_{c}$, can be written as follows:

(6) $$E_{c}=E_{g}+\sum p_{i}\left(E_{i}-E_{g}\right)$$

where $E_{c}$ and $E_{g}$ are the potential energies of the combined and ground/stable states, respectively, and $p_{i}$ and $E_{i}$ the weight and potential energy of metastable state *i*. 

In Figure 2a, the equilibrium separation distance of the metastable state is smaller than that of the ground state, and their potential energy curves cross each other at $r_{ig}$. The combination of the metastable state with the ground state thus increases the energy of the ground state at the separation distances larger than $r_{ig}$, and decreases the energy at the separation distances smaller than $r_{ig}$. The equilibrium separation distance of the combined state is thus smaller than that of the ground state though with a higher potential energy. In case the potential energy curves of two states do not cross each other, the addition of the metastable state increases the energy of the ground state more at the larger separation distance than at the smaller separation distance, and still decreases the equilibrium separation distance of the combined state. On the other hand, Figure 2b shows the case that the equilibrium separation distance of the metastable state is larger than that of the ground state. The combined state thus has a larger equilibrium separation distance than the ground state.

Mathematically, it is then possible to find a set of metastable states so that the combined potential energy represented by Equation 6 becomes quadratic (Figure 1b) first and then tilts to smaller separation distance (Figure 1c) at the vicinity of its new equilibrium separation distance. The potential energy thus becomes symmetric with zero thermal expansion or exhibits anomaly with thermal contraction. In the next section, energetics of ground and metastable states and their weights at finite temperatures will be discussed and evaluated in terms of DFT-based first-principles calculations and statistical mechanics. Instead of the separation distance used in this section for the sake of simplicity, the volume is used in the rest of the paper.

It should be mentioned that Barrera et al. [4] discussed the concept of “apparent” and “true” separation distances for the distance between the mean positions of two atoms and the mean distance between two atoms, respectively. The “apparent” separation distance is obtained from X-ray or neutron diffraction measurements with low temporal resolution, and the “true” separation distances from extended X-ray absorption fine structure (XAFS) with high temporal resolution. One may attempt to connect the “apparent” and “true” separation distances with the separation distances of the combined state and the individual states in the present work, respectively. This was demonstrated in our recent *ab initio* molecular dynamic simulations of PbTiO_3_ in comparison with the experimental X-ray and XAFS data in the literature showing the apparent cubic structure by X-ray and neutron diffraction measurements and the significant amount of “true” local tetragonal structures by XAFS [16]. 

## 4. Statistical Mechanics of Ground and Metastable States and Their Interactions

**Figure 3:**
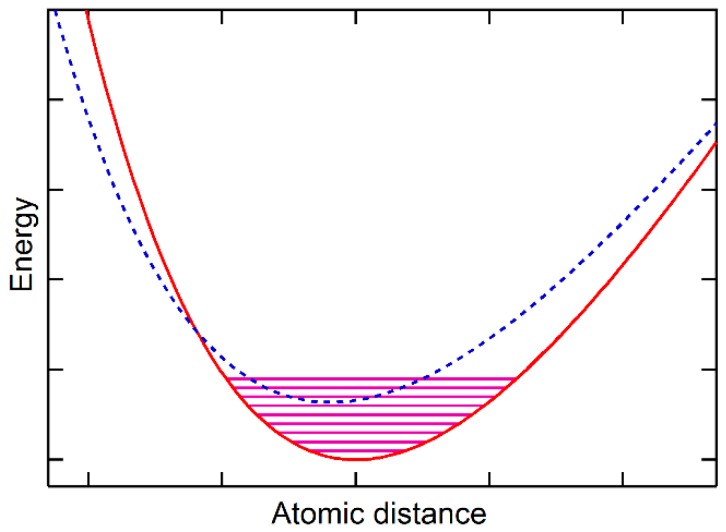
Schematic diagram showing the ground state and a metastable state with the vibrational energy of the ground state reaching the potential energy of the metastable state.

At a given temperature and pressure (zero external pressure used herein for simplicity), the equilibrium volume of a state *i*, $V_{i}$, of a substance is determined when its Helmholtz energy, defined as $F_{i}=E_{i}-TS_{i}$, is minimized, where the entropy of the state, $S_{i}$, is typically obtained from quasi-harmonic first-principles phonon calculations or Debye models including both vibrational and thermal electronic contributions [17,18,19,20]. It should be noted that the phonon calculations are usually based on the potential energy at 0 K. In the cases where the vibrational energy of the ground state reaches the potential energy of metastable states, the calculated vibrational energy of the substance contains thus vibrational contributions from both the ground state and the metastable state at temperatures close to 0 K, representing the quantum origin of the vibrational energy for thermal expansion. With the metastable state having smaller volumes than the ground state, as schematically shown in Figure 3, the substance can show thermal contraction as demonstrated for ice and Si close to 0 K in our previous publication [10] and in the literature [21], representing the quantum origin of the vibrational energy for thermal contraction. The rest of the paper addresses cases when the vibrational energy of the ground state is smaller than the energy difference between the ground state and the metastable states, i.e., typical phonon calculations do not show property anomalies.

In the canonical ensemble with *N*, *V*, and *T* as thermodynamic natural variables, the canonical partition function of a state is written as

(7) $$Z_{i}=e^{-\frac{F_{i}}{k_{B}T}}=\sum_{k}e^{-\frac{E_{ik}}{k_{B}T}}$$

where $E_{ik}$ are the energy eigenvalues of microstate *k* in the state *i*, including the contributions from phonons and thermal electrons [12]. For a system with multiple states schematically shown in Figure 2, the canonical partition function of the system is as follows [8,9,22]:

(8) $$Z_{c}=e^{-\frac{F_{c}}{k_{B}T}}=\sum_{j}e^{-\frac{E_{cj}}{k_{B}T}}$$

where $F_{c}$ is the Helmholtz energy of the system with multiple states, and $E_{cj}$ the energy eigenvalues of microstate *j* in the combined state, *c*, including contributions from phonons and thermal electrons [9,12]. The contribution of each state to the combined state, i.e., the weights in Equation 6, can be defined by the partition functions as follows:

(9) $$p_{i}=\frac{Z_{i}}{Z_{c}}.$$

Combining Equations (8) and (9) and rearranging them, one obtains [8,9,10,22]

(10) $$F_{c}=-k_{B}TlnZ_{c}=-k_{B}T\left(\sum \frac{Z_{i}}{Z_{c}}lnZ_{i}-\sum \frac{Z_{i}}{Z_{c}}ln\frac{Z_{i}}{Z_{c}}\right)=\sum p_{i}F_{i}-TS_{SCE}$$

with $S_{SCE}=-k_{B}\sum p_{i}lnp_{i}$ defined as the state configurational entropy [10]. The total entropy of the system of the combined state can then be written as

(11) $$S_{c}=\sum p_{i}S_{i}+S_{SCE}.$$

It is important to realize that the entropy of the system with the combined potential energy is not a simple weighted summation of entropies of individual stable and metastable states, but contains an addition contribution representing statistical competition among the stable and metastable states. This contribution to the total entropy helps to bring those metastable states not accessible in terms of typical phonon calculations at 0 K into the statistical existence at high temperatures, which in turn introduce property anomalies that the ground and metastable states do not possess individually. Another important observation from Equation (10) is that $F_{c}$ can either be calculated from the combined state using Equation 8 or from the individual states using Equation (7) and the last part of Equation (10), and they are connected by Equation (9).

Let us further discuss the situation presented in Figure 2a, where the potential energies of the ground and metastable states cross each other at a volume smaller than that of the ground state and their energy difference is larger than phonon energy of the ground state, i.e., $p_{i}\approx 0$ for the metastable state at low temperatures. The two states are thus in equilibrium with each other at 0 K at the pressure equal to the negative of the common tangent of the two potential energy curves. For the metastable state to become part of the combined state, its weight, $p_{i}$ as defined by Equation (9), must increase with temperature, i.e., the entropy of the metastable state must be higher than that of the ground state. This equilibrium line between the state *g* and the state *i*, which is now also a two-phase equilibrium line in the temperature–pressure space, thus has a negative slope based on the Clausius–Clapeyron relation as discussed in our previous work [2,10]:

(12) $$\frac{dP}{dT}=\frac{∆S}{∆V}<0$$

This is the necessary condition for the ground state phase to exhibit thermal contraction in the single-phase region of the ground state, *g*, but this is not a sufficient condition. There can be at least two scenarios in which the thermal contraction may not be observed. The first one is where the volumes increase due to the thermal expansions of individual stable and metastable states is more significant than the volume difference between the states, resulting in the net volume increase with temperature instead of decrease though in a smaller scale than the single state *g*. The second one is the existence of additional metastable states with larger equilibrium volumes than that of the ground state, as shown in Figure 2b, and comparable values of $p_{i}$, resulting in again the net volume increase with temperature.

As mentioned above, a metastable state may never be in equilibrium with the ground state at any pressures if their potential energy curves do not cross each other. Since this condition is not required in the present theory, the metastable state may still introduce anomalies in the combined state if their contribution becomes significant enough.

## 5. Discussions

The theory presented in the paper indicates that the thermal contraction at temperatures close to 0 K can be directly predicted from phonon calculations of the ground state, which was demonstrated for ice and silicon [10]. For thermal expansion anomalies at high temperatures, both the infinite thermal expansion and infinite thermal contraction were predicted for cerium [8,22] and Fe_3_Pt [10] at their respective critical points, and the anomalies in the single-phase region away from critical points using the theory described in the present paper.

It is evident from Equation 6 that the combined potential energy can have contributions from more than one metastable states. That was the qualitative application of our theory to the ZrW_2_O_8_ system [2]. The temperature–pressure phase diagram was proposed by Arora et al. [23] with the α- ZrW_2_O_8_ phase stable at low pressures and low temperatures, the β-ZrW_2_O_8_ phase stable at low pressures and high temperatures, the γ-ZrW_2_O_8_ phase stable at high pressures and low temperatures, plus an amorphous phase at higher pressures, and the amorphous phase at high pressures/low temperatures and low pressures/high temperatures, respectively. The two-phase equilibrium line between γ/amorphous is negative as the amorphous phase has higher entropy and smaller volume than the γ phase, resulting in thermal contraction in the γ single phase [24] in accordance with our theory. However, the α/γ and β/γ two-phase lines are both slightly positive. If there were only these phases, both α and β phases would have shown thermal expansion. However, due to the existence of the amorphous phase, the metastable α/amorphous and β/amorphous two-phase lines should also be negative, which is also shown by the negative slope of the stable β/amorphous two-phase line at high temperatures, resulting in thermal contraction in both single α and single β phase regions [25]. 

The recent review by Dove and Fang [7] mentioned that our theory does not apply to the thermal contraction observed in cubic ScF_3_ because the two-phase equilibrium line between its cubic and rhombohedral structures at high pressures is positive in the ranges of pressure and temperature up to 0.6 GPa and 300 K, respectively [26]. However, what Dove and Fang might not be aware is that ScF_3_ transforms to an amorphous phase at 3.8 GPa shear deformation [27]. Even though this pressure is higher than the 1.5 GPa pressure for the formation of amorphous ZrW_2_ O_8_, it is highly possible that this amorphous ScF_3_ phase contributes to the thermal contraction behavior of cubic ScF_3_ due to the smaller volume and higher entropy of the amorphous phase, in accordance with our theory and similar to the α- and β-ZrW_2_O_8_ phases. Furthermore, our theory indicates that the rhombohedral ScF_3_ should also show the thermal contraction behavior in a wide range of temperature and pressure due to its lower entropy and larger volume than those of amorphous ScF_3_, which has not been reported in the literature. 

**Figure 4:**
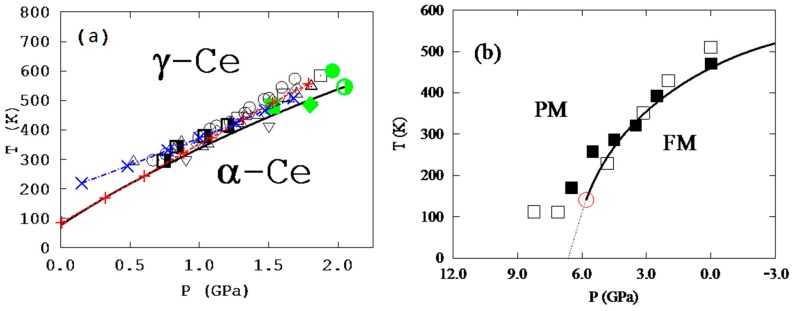
Predicted temperature–pressure phase diagrams: (**a**) cerium adapted from [22], with permission from © 2009 IOP Publishing, and (**b**) Fe_3_Pt adapted from [9], with permission from © 2010 Taylor & Francis.

One challenge in the application of our theory is to sample all important states with their potential energy curves close to that of the ground state as shown in the figures and implied by Equations (6) and (9). In the calculations of cerium, two states, i.e., non-magnetic ground state and ferromagnetic metastable state, were first tested, and a mean-field magnetic contribution was added separately to the magnetic state as commonly done in the literature [8,10]. The temperature–pressure phase diagram of fcc cerium with a critical point and a miscibility gap was then predicted, showing excellent agreement with all experimental data for the first time along with predictions of temperature ranges of colossal thermal expansion for various pressures in the single-phase region. To further improve the model, one additional anti-ferromagnetic state was introduced [22], and it was found that the mean-field magnetic contribution term was not needed, with similar agreement between predictions and experiments. The predicted temperature–pressure phase diagram is shown in Figure 4a. This is most likely because the magnetic disordering is effectively included when the anti-ferromagnetic state is added into the ensemble, and this state contains the interactions of opposite magnetic spins, i.e., the effects of magnetic domain wall.

For Fe_3_Pt, a supercell with 9 Fe atoms was used, giving 2^9^ = 512 states of different magnetic spin configurations [9,10], among which 37 are non-equivalent ones due to symmetry. The first-principles calculations showed that the ferromagnetic state is the most stable one at all temperatures considered, up to 900 K, but the Helmholtz energy differences between the stable and metastable states decrease with increasing temperature [2], which increases $p_{i}$ of the metastable states with their sum over 90% at 900 K, a fully paramagnetic state. The temperature–pressure phase diagram of Fe_3_Pt was then predicted with a critical point and a miscibility gap as shown in Figure 4b. Again, energetics of domain walls are effectively considered in the 37 independent spin flipping states with two of them with lowest energies amounting to more than 80% of the all metastable states.

**Figure 5:**
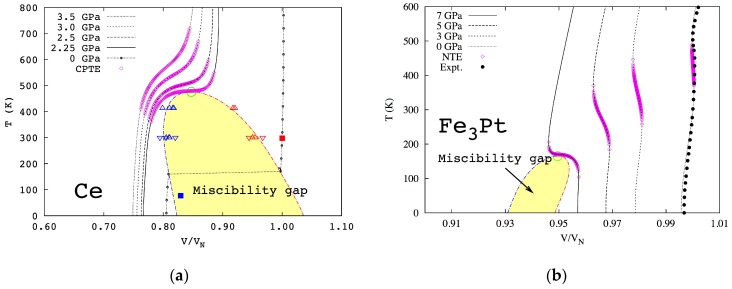
Predicted temperature–volume phase diagrams adapted from [10], with permission from © 2014 Nature Publishing Group, (**a**) cerium and (**b**) Fe_3_Pt.

As is expected, the slope of the two-phase equilibrium line in Fe_3_Pt below the critical point is negative [9,10] in contrast to the positive slope in cerium [22]. To better visualize the thermal expansion behaviors, the pressure is replaced by its conjugate variables volume to obtain the temperature–volume phase diagrams as shown in Figure 5 for cerium and Fe_3_Pt, respectively, with the volume normalized to its equilibrium volume at atmospheric pressure and room temperature. Below the critical point marked by the green open circles, the single phase is no longer stable and decomposes into a two-phase mixture in the area of miscibility gap. The isobaric volumes of cerium and Fe_3_Pt are plotted for various pressures, respectively. The predicted thermal expansion/contraction regions are illustrated by the pink open diamonds. It can be seen that the thermal expansion/contraction anomaly exists in regions far away from the critical points.

The isobaric curve in Figure 5a under 2.25 GPa shows that the volume increases with temperature normally at low temperatures, and the increasing rate becomes much higher at higher temperatures and reaches the maximum at the critical point before reducing back to normal. With increasing pressure, the isobaric curve moves away from the critical point, and the maximum thermal expansion decreases. In the case of Fe_3_Pt, the volume also increases normally with temperature at low temperatures, but the increasing rate decreases at higher temperatures and becomes zero at a certain temperature and negative above that temperature, reaching the most negative value at the critical point under 7 GPa and then becoming less negative, zero, and normal positive at higher temperatures. At lower pressures, the phenomenon persists though with lower maximum negative thermal expansion. This sequence is in excellent agreement with our theory presented in Figure 1, Figure 2 and Figure 3.

The theory presented in this paper has been used for a number of systems including Ni [28], Fe [29], BaFe_2_As_2_ [30], EuTiO_3_ [31], and Cu_2_ZnSn(S,Se)_4_ [32], and it has also been developed to treat the unstable vibrational mode of transition states in solid-state diffusion [33]. For a more general search of stable and metastable states of a given structure, the ATAT [34] and USPEX [35] packages are being tested in our research activities. The databases of Helmholtz energy functions of important stable and metastable states of substances as a function of temperature, pressure/strain, composition, and electric/magnetic fields may thus serve as materials genome databanks and be used to discover and design new materials [36]. Furthermore, in the first-principles calculations, the supercell structures and sizes need to be tested to show convergence of the results.

## 6. Conclusions

The fundamental thermodynamics of thermal expansion and contraction is revisited in this paper. It is shown that a complex potential energy of a system can be constructed by the weighted summation of potentials of ground and metastable states. It is demonstrated that statistical mechanics, with the weights of individual states defined by the ratio of the partition functions of individual states to the combined state, can quantitatively predict the critical point and miscibility gap in cerium and Fe_3_Pt and their respective colossal thermal expansion and thermal contraction and qualitatively explain the observations of thermal contraction in ZrW_2_O_8_, ScF_3_, and other substances. It is anticipated that the theory presented in the paper can be used to predict a wide range of properties at finite temperatures.
